# A Case of Acute-Onset, Refractory Freezing of Gait Following Subthalamic Nucleus Deep Brain Stimulation Adjustment

**DOI:** 10.7759/cureus.89437

**Published:** 2025-08-05

**Authors:** Brendan Baugher, Ellen Walter, Erica Hennigs, Kristen Appleby, Benjamin L Walter, James Liao

**Affiliations:** 1 Center for Neurological Restoration, Neurological Institute, Cleveland Clinic, Cleveland, USA

**Keywords:** deep brain stimulation, fog, freezing of gait, parkinson’s disease, subthalamic nucleus

## Abstract

Freezing of gait (FoG) is a disabling symptom of Parkinson’s disease (PD) characterized by involuntary cessation/reduction. While deep brain stimulation (DBS) targeting the subthalamic nucleus (STN) effectively treats common PD symptoms such as tremor, its impact on FoG is less clear. Rarely, STN-DBS itself can induce FoG. Reversible cases have been linked to hyperdopaminergic states, high-frequency stimulation, and suboptimal DBS lead placement. One *irreversible* case occurred immediately after DBS surgery and was attributed to lesioning of gait pathways during lead insertion. We report a case of seemingly irreversible FoG that began not after lead insertion or initial activation, but after initial follow-up *adjustment *in STN-DBS with otherwise proper placement and expected benefit.

A 62-year-old female underwent STN-DBS for medication-resistant symptoms of idiopathic PD. The leads were activated at 125 Hz without adverse events. The patient returned two weeks later for stimulation adjustment. Two days after that visit, the patient developed severe FoG, resulting in falls. Despite many adjustments to both stimulation parameters and medications, the patient continued to experience FoG even with the device off. Lower frequency stimulation (55 Hz) provided partial, temporary improvement in FoG. Low-frequency parameters and off-stimulation trials were limited by the patient’s severe dystonia. Spatial reconstruction confirmed the active contacts were within the STN.

This timeline of events differs from previously reported cases attributed to anatomic/structural causes (lead mispositioning vs. gait pathway lesioning), hyperdopaminergic states, or high-frequency stimulation. Although the utilized contacts appear to be appropriately positioned, iatrogenic induction of FoG is too poorly understood to exclude their position as a factor. This case could be explained as a combination of positional- and frequency-induced FoG if not for the persistence of FoG when off-stimulation. It is possible that the “off trials” were not long enough for the adverse effects of stimulation to fully subside. Longer “off trials” are limited by the patient’s severe dystonia.

Post-STN-DBS FoG refractory to stimulus on/off state is a rare phenomenon reported only twice in the current literature (including this case). The current report describes the first patient for whom significantly refractory FoG specifically began after stimulus adjustment; this may represent lasting negative effects of stimulation in the DBS “off” state. Comprehensive reporting of anatomical lead locations and stimulation parameters in similar cases is essential to identify patterns that could inform future interventions.

## Introduction

Freezing of gait (FoG) is a common yet enigmatic symptom of Parkinson’s disease (PD) characterized by involuntary cessations or reductions in ambulation despite the intent to walk [1]. These “freezes” are a source of significant anxiety and injury for the PD population, reducing independence and quality of life. Despite many theories, its pathophysiology remains poorly understood. Its limited response to dopaminergic medications and deep brain stimulation (DBS) has generated a reputation among clinicians as being challenging to treat [2].

The subthalamic nucleus (STN) is a well-established target for DBS, effective in treating PD symptoms such as tremor, dystonia, and bradykinesia [3]. Effects of STN-DBS on FoG are less predictable [4]. Though uncommon, significant worsening and even new-onset FoG have been observed after STN-DBS. Months to years after DBS activation, new on-medication FoG has been described when relatively high doses of dopaminergic medications were used in conjunction with STN-DBS for refractory symptoms [5,6]. The FoG in these cases notably improved when each respective therapy was used alone [6] or lower doses of dopaminergic medications were used [5], suggesting a potential threshold of dopaminergic support beyond which gait dysfunction is induced or worsens.

Acute onset of FoG after STN-DBS has also been described. New FoG has been reported immediately on activation of DBS leads at 130 Hz and resolving with reduction of stimulation frequency to 60 Hz [7]. Lower frequencies are thought to be more effective for FoG/gait dysfunction [3,8,9]. Other cases of acute post-DBS FoG suggest lead placement/positioning may be culpable in inducing FoG. One patient with off-medication FoG developed on-stimulation FoG immediately after lead activation that resolved with cessation of stimulation [10]. MRI revealed their left lead was more anteromedial in the STN than in the planned trajectory; FoG symptoms resolved with surgical revision. It was hypothesized that this errant stimulation had disrupted signaling pathways between the pedunculopontine nucleus (PPN) and the basal ganglia. The PPN is one key component of the mesencephalic locomotor region (MLR), which is instrumental in gait control [11]. Its role in FoG is still under investigation, as illustrated by a recent negative trial targeting the PPN in DBS for FoG [12].

An incidence of new-onset, post-STN-DBS FoG that had little to no response to medications or changes/cessation in stimulation has also been described [13]. Interestingly, this FoG developed in the immediate postoperative period, before stimulation was started. It was hypothesized that the insertion of DBS leads had lesioned PPN-related pathways, triggering a severe FoG phenotype. We report the second case of seemingly irreversible post-STN-DBS FoG. In this patient, FoG emerged shortly after their first post-activation adjustment and subsequently exhibited resistance to both stimulation and medication state.

## Case presentation

A 62-year-old female with a five-year history of idiopathic PD was evaluated as being a suitable candidate for bilateral STN-DBS due to medication-resistant and wearing-off symptoms. Her symptoms included right-sided rigidity and bradykinesia, REM sleep disorder, and, most bothersome, painful left toe dystonia. She had no history of FoG, tremor, or dyskinesia, and no pertinent comorbidities. Preoperative PD medications included Rytary (carbidopa-levodopa extended release) 23.75-95 mg capsules on a 4-5-4-3 schedule (number of capsules in early morning-late morning-early afternoon-evening) and Requip (ropinirole) XL 6 mg daily.

**Figure 1:**
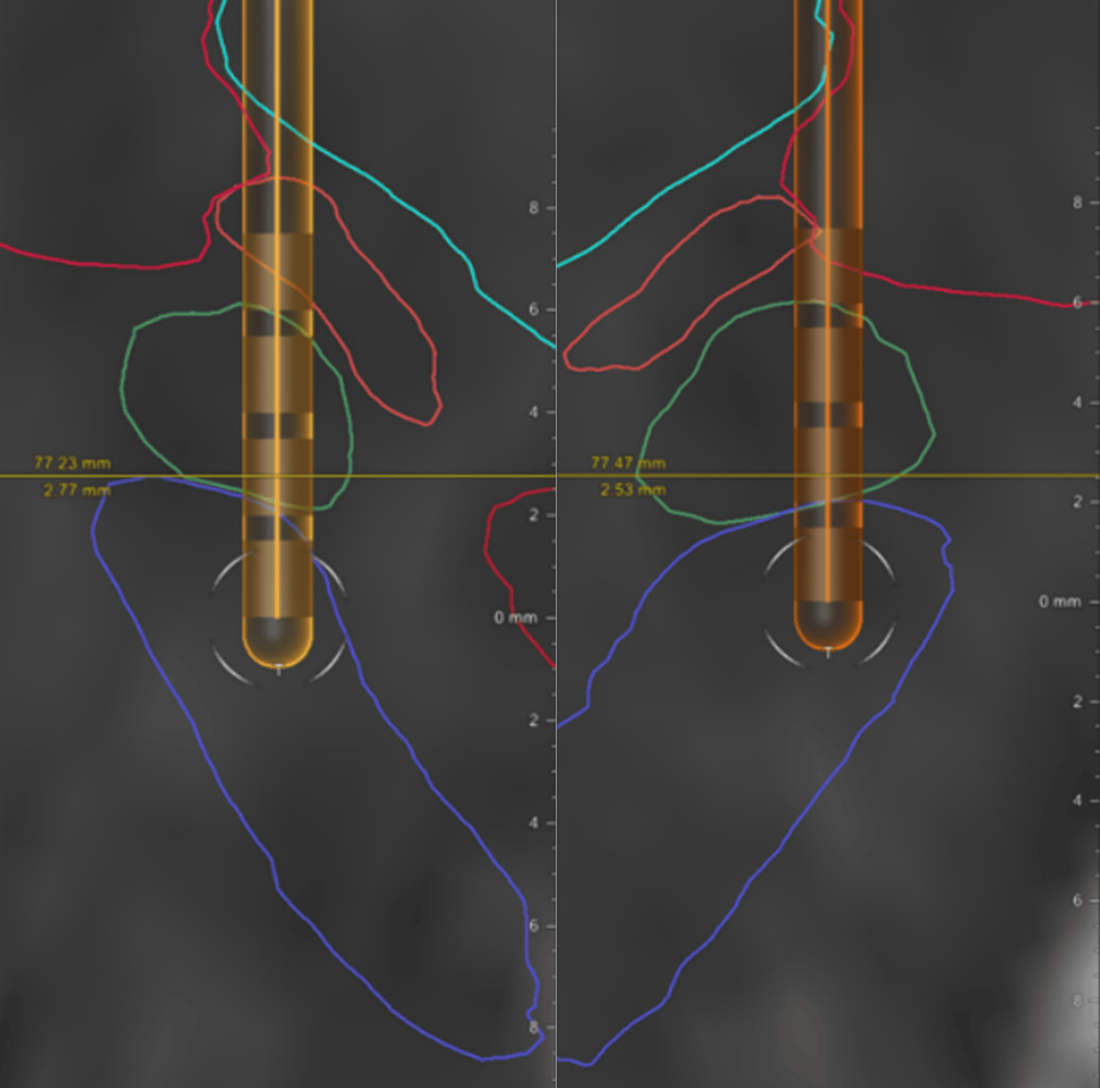
Post-surgical, posterior inline (image plane perpendicular to the electrode trajectory) T2 MRI with spatial reconstruction of left (left) and right (right) DBS leads. The subthalamic nuclei are outlined in green, the substantia nigra in dark blue/purple, the zona incerta in orange, the thalami in light blue, the internal capsules (large structures in top corners) in pink, and the red nucleus in red.

A Medtronic Sensight (model B33015) DBS system was implanted in stages: the left lead, followed by the right lead fifteen days later, and the pulse generator nine days after that. Microelectrode physiology was used to place the top edge of contacts 2 and 10 just under the dorsal edge of the STN. The patient and providers noted no unanticipated adverse events to that point. Postoperative imaging with spatial reconstruction of the leads using Brainlab (Munich, Germany) software suggested successful implantation with the two central contacts of each lead within the STN (Figure 1). On the left, from inferior to superior, contact 0 lies within the substantia nigra (SN), contact 1 in the ventral STN (abutting the SN), contact 2 in the posterodorsal STN, and contact 3 is just superior to the STN, partially within the zona incerta. On the right, from inferior to superior, contact 8 lies within the SN, contact 9 in the ventrolateral STN, contact 10 in the dorsolateral STN, and contact 11 superior to the STN between the zona incerta and internal capsule.

The leads were activated at an initial programming session one month after battery placement. At the second programming visit, 12 days after the first, the patient reported development of dyskinesias and “imbalance” on the initial settings. A new configuration with a different active contact on the right (contact 10 instead of contact 9) appeared to alleviate these symptoms in the office.

Approximately three weeks after this second programming visit, the patient called the movement disorders clinic to communicate that, two days after the second session (which was six weeks post-battery placement, two weeks post-lead activation), she had developed severe episodes of FoG and shuffling gait several times per day. These episodes, which were particularly problematic during turns, occurred multiple times daily and led to several falls, one of which resulted in a humeral head fracture. Notably, no change in symptoms was observed with different stimulation groupings, varying stimulation amplitudes within the provided range, or even turning the device off entirely. No temporal pattern in relation to their medications was noted, either.

Over the next 18 months, the patient returned for a total of 14 programming sessions to address the FoG and other PD symptoms (Table 1). Numerous DBS adjustments and medication regimen changes were attempted, with little lasting impact on FoG symptoms. As seen in Table 1, some medication changes were also driven by issues with insurance coverage. During each visit, both the examiner and the patient felt that the DBS adjustments seemed to improve FoG, step length, and/or turning in the clinic. Recurrently, though, the patient would return with complaints of FoG disrupting her quality of life, causing falls and, understandably, contributing to worsening mood symptoms.

**Table 1:** Notable deep brain stimulation follow-up visits.* Each “Group” refers to grouped stimulator settings available to the patient; four Groups were utilized (A-D); unlisted groups were inactive at the time of the visit; stimulation parameters listed as “left lead, right lead” or a single value if both leads were on the same setting. *: This is a summary of the most notable DBS clinic follow-up visits, not an exhaustive list of interactions with the patient over this period. Med(s) = medication(s); C+ = case positive; Amp = amplitude in mA; PW = pulse width in μ-seconds; F = frequency in Hz; ↑ = increased; ↓ = decreased; ↔ = persistent

Appointment (weeks since battery placement; since lead activation)	On arrival	Leaving visit
1^st^ programming visit [4;0]	Groups: None, leads inactive	Group A (active): 1-/C+, 9-/C+; Amp 1.5, 1.7; PW 60; F 125
	Meds: Rytary 23.75–95 mg 4-5-4-2, Requip XL 6 mg	Group B (backup): 2-/C+, 9b/C+; Amp 1.5, 0.9; PW 60; F 125
	Symptoms: Rigidity, bradykinesia, left toe dystonia	Med changes: None
	-	Observations: Group A ↓rigidity, bradykinesia, left toe dystonia
2^nd^ programming visit [6;2]	Group A (active): 1-/C+, 9-/C+; Amp 1.5, 1.7; PW 60; F 125	Group C (active): 1-/C+, 10a-/C+; Amp 1.7, 2.1; PW 60; F 125
	Group B (backup): 2-/C+, 9b-/C+; Amp 1.5, 0.9; PW 60; F 125	Group A (backup): 1-/C+, 9-/C+; Amp 1.7, 0.8; PW 60; F 125
	Meds: Rytary 23.75–95 mg 4-5-4-2, Requip XL 6 mg	Group B (backup): 2-/C+, 9a-/C+; Amp 1.5, 2.6; PW 60; F 125
	Symptoms: Using Group A exclusively, ↑dyskinesias and “imbalance”	Med changes: Rytary 23.75–95 mg 3-2-3-2, Requip XL 6 mg
	-	Observations: Group C ↑balance and ↓bradykinesia
3^rd^ programming visit [10;6]	Group C (active): 1-/C+, 10a-/C+; Amp 1.7, 2.1; PW 60; F 125	Group B (active): 1-/C+, 10-/C+; Amp 1.1, 1.2; PW 60; F 125
	Group A (backup): 1-/C+, 9-/C+; Amp 1.7, 0.8; PW 60; F 125	Group A (backup): 1-/C+, 9-/C+; Amp 1.7, 0.8; PW 60; F 125
	Group B (backup): 2-/C+, 9a-/C+; Amp 1.5, 2.6; PW 60; F 125	Group C (backup): 1-/C+, 10b-/C+; Amp 1.9, 2.3; PW 60; F 125
	Meds: Rytary 23.75–95 mg 3-3-3-3, Requip XL 6 mg	Med changes: None
	Symptoms: Developed FoG two days after the second session; ↑falls; no groups or turning off DBS ↓symptoms	Observations: Group B ↑stride length and improved turns
4^th^ programming visit [14;10]	Group A (active): 1-/C+, 9-/C+; Amp 1.9, 1.0; PW 60; F 125	Group D (active): 2a-/C+, 9b-/C+; Amp 1.5, 1.9; PW 60; F 125
	Group B (backup): 1-/C+, 10-/C+; Amp 1.1, 1.2; PW 60; F 125	Group A (backup): 1-/C+, 9-/C+; Amp 1.9, 1.0; PW 60; F 125
	Group C (backup): 1-/C+, 10b-/C+; Amp 1.9, 2.3; PW 60; F 125	Group B (backup): 1-/C+, 10-/C+; Amp 1.1, 1.2; PW 60; F 125
	Meds: Rytary 23.75–95 mg 3-3-3-3, Requip XL 6 mg	Group C (backup): 1-/C+, 10b-/C+; Amp 1.9, 2.3; PW 60; F 125
	Symptoms: Using Group A as she felt falls/balance were worse with the others; ↔ FoG	Med changes: None
	-	Observations: Group D ↓FoG in clinic
Non-programming visit [16.5;12.5]	Group A (active): 1-/C+, 9-/C+; Amp 1.9, 1.1; PW 60; F 125	Group A (active): 1-/C+, 9-/C+; Amp 1.9, 1.1; PW 60; F 125
	Group B (backup): 2-/C+, 10-/C+; Amp 1.1, 1.2; PW 60; F 125	Group B (backup): 2-/C+, 10-/C+; Amp 1.1, 1.2; PW 60; F 125
	Group C (backup): 1-/C+, 10b-/C+; Amp 1.9, 2.3; PW 60; F 125	Group C (backup): 1-/C+, 10b-/C+; Amp 1.9, 2.3; PW 60; F 125
	Group D (backup): 2a-/C+, 9b-/C+; Amp 1.5, 1.9; PW 60; F 125	Group D (backup): 2a-/C+, 9b-/C+; Amp 1.5, 1.9; PW 60; F 125
	Meds: Rytary 23.75–95 mg 3-3-3-3, Requip XL 6 mg	Med changes: Rytary 23.75–95 mg 3-5-3-2, started amantadine 100 mg BID
	Symptoms: ↔FoG, no group ↓FoG but C ↑FoG; improvement in other PD symptoms since surgery	Observations: Started PD-specific physical therapy
5^th^ programming visit [18.5;14.5]	Group A (active): 1-/C+, 9-/C+; Amp 1.8, 1.0; PW 60; F 125	Group B (active): 1-/C+, 2c-/C+ and 9-/C+; Amp 1.8, 1.0; PW 60; F 125
	Group B (backup): 2-/C+, 10-/C+; Amp 1.1, 1.2; PW 60; F 125	Group A (backup): 1-/C+, 9-/C+; Amp 1.8, 1.0; PW 60; F 125
	Group C (backup): 1-/C+, 10b-/C+; Amp 1.9, 2.3; PW 60; F 125	Group C (backup): 1-/C+, 10b-/C+; Amp 1.9, 2.3; PW 60; F 125
	Group D (backup): 2a-/C+, 9b-/C+; Amp 1.5, 1.9; PW 60; F 125	Group D: Discontinued
	Meds: Rytary 23.75–95 mg 3-5-3-2, Requip XL 6 mg, amantadine 100 mg BID	Med changes: None
	Symptoms: ↔FoG and falls	Observations: ↓FoG with new Group B
6^th^ programming visit [22;18]	Group B (active): 1-/C+ and 2c-/C+, 9-/C+; Amp 1.8, 1.0; PW 60; F 125	Group C (active): 1a-/C+ and 2a-/C+, 9b-/C+ and 10b-/C+; Amp 1.6, 1.8; PW 60; F 125
	Group A (backup): 1-/C+, 9-/C+; Amp 1.8, 0.8; PW 60; F 125	Group A (backup): 1-/C+, 9-/C+; Amp 1.8, 1.1; PW 60; F 125
	Group C (backup): 1-/C+, 10b-/C+; Amp 1.9, 2.3; PW 60; F 125	Group B (backup): 1-/C+ and 2c-/C+, 9-/C+; Amp 2, 1.4; PW 60; F 125
	Meds: Rytary 23.75–95 mg 3-5-3-2, Requip XL 6 mg, amantadine 100 mg BID	Med changes: Rytary 23.75–95 mg 2-3-2-2
	Symptoms: ↔FoG and falls; ↑ dyskinesias with A and B	Observations: ↑gait stability but ↔FoG with Group C
9^th^ programming visit [36;32]	Groups: Unclear, F in all set at 125 Hz	Group B: F↑ to 180 Hz
	Meds: Rytary 23.75–95 mg 3-3-2-2, Requip XL 6 mg, amantadine stopped one month prior for inefficacy	Med changes: None
	Symptoms: ↔FoG, ↑falls/anxiety; other symptoms (tremor, rigidity, dyskinesias) controlled	
10^th^ programming visit [45;41]	Group B: 2b-/C+, 9-/C+; Amp 2, 2.8; PW 90; F 180	Group B: 2b-/C+, 9-/C+; Amp 2, 2.1; PW 70, 60; F 80
	Meds: Sinemet IR 25–100 mg 2-2-2-2 (Rytary stopped due to insurance issues), Requip XL 6 mg	Med changes: None
	Symptoms: ↔FoG and falls, no change with increased frequency	Observations: ↓FoG with ↓frequency
11^th^ programming visit [60;56]	Group B: 2b-/C+, 9-/C+; Amp 2, 2.1; PW 70, 60; F 80	Group C (active): 2b-/C+, 9b-/C+; Amp 3.5, 3.3; PW 70, 60; F 55
	Meds: Rytary 23.75–95 mg 3-4-2-2, Requip stopped two weeks earlier due to insurance issues	Group B (backup): 2b-/C+, 9b-/C+; Amp 2.3; PW 70, 60; F 80
	Symptoms: ↔FoG with lower frequency	Observations: ↓FoG(but not resolution) with ↓frequency
13^th^ programming visit [93;89]	Group A (active): 2b-/C+, 9b-/C+ and 10b-/C+; Amp 3, 2.55; PW 70, 60; F 55	Group A (active): 2b-/C+, 9b-/C+ and 10b-/C+; Amp 3, 2.75; PW 70, 60; F 55
	Group B (backup): 2b-/C+, 9b-/C+; Amp 3.5, 3.9; PW 70, 80; F 55	Med changes: None
	Group C (backup): 2b-/C+, 9b-/C+; Amp 3.5, 4; PW 70, 60; F 55	Observations: Severe FoG/falls at ↑Amp/F vs dystonia/dyskinesias/↓FoG at ↓Amp/F
	Meds: Rytary 23.75–95 mg 3-4-2-2	
	Symptoms: ↓DBS lead to ↓FoG but ↑left toe dystonia and vice versa; ↔FoG with DBS off	

Documentation suggests that the patient’s Rytary dose was decreased around the time of the visit, just before FoG onset (from a 4-5-4-2 schedule to 3-2-3-2). However, it appears that the patient was taking fewer tablets than documented, as the provider noted the change to a strict 3-2-3-2 schedule would represent an increase in medication and was concerned that the patient was “undermedicated” at the time. Regardless, subsequent increases/decreases in Rytary/Sinemet had no reported impact on FoG. This included a lack of improvement in FoG with Sinemet IR 25-100 mg 2-2-2-2, which was a similar L-dopa equivalent daily dose (LEDD) to the “original” Rytary dosing.

The most significant stimulus-related change during the visit immediately preceding the onset of FoG was a switch from contact 9 to contact 10 in the right lead. Based on the spatial reconstruction imaging, this corresponded to a shift in stimulus focus from the ventrolateral to dorsolateral STN. This was discussed as a potential instigating factor of the refractory FoG, yet switching back to the ventrolateral STN contact later did not seem to improve symptoms.

Both high- and low-frequency stimulation were trialed. Higher frequencies, as high as 180 Hz, controlled the patient’s non-FoG symptoms, including dyskinesias and painful toe dystonia. Surprisingly, high frequencies had no clear effect on freezing frequency or severity. One year after DBS implantation, FoG was reportedly the patient’s most bothersome symptom; hence, lower frequencies (down to 55 Hz) were tested. This change was the only one noted to produce a sustained improvement in FoG for several weeks; however, even during this period, the patient experienced several falls during freezing episodes. Ultimately, the benefits of lower rates on FoG seemed to diminish, and the patient experienced increasingly disruptive dystonia at lower stimulation levels. This pattern, an initial benefit of lower frequencies on FoG that dissipates in the short term, and a subsequent return to higher frequencies for adequate coverage of other symptoms, has been described previously in STN-DBS [14].

Currently, FoG remains a major challenge for the patient, often preventing her from engaging in activities outside the home and causing substantial social and fall-related anxiety. She maintains that her other PD symptoms have improved since before the DBS surgery, but hopes to find a treatment to help manage or mitigate her FoG. In this pursuit, she continues to work with her neurologic care team and engage in PD and FoG-specific physical therapy.

## Discussion

Previous cases of post-STN-DBS FoG can be broadly categorized by three hypothesized mechanisms, namely, anatomic/structural, high-frequency stimulation, and hyperdopaminergic states. These hypotheses range from speculative to well-established. Cases related to mechanical disruption or high frequencies typically present acutely, while hyperdopaminergic-induced FoG tends to emerge later, as disease progression necessitates higher medication doses and stimulation parameters [5,6]. The present case shares features with several prior reports but does not align neatly with any of these categories.

Unintended anatomic/structural causes have been implicated in two prior cases of post-STN-DBS FoG. One involved mispositioning of leads within the STN, causing immediate FoG upon activation [10]. The other was suspected to be secondary to structural lesioning of PPN-related tracts during implantation, leading to irreversible FoG even off stimulation, like in the present case [13]. Unlike that lesioning case, our patient’s persistent FoG did not begin immediately after surgery, but after the settings were initially adjusted. This would suggest that mistargeted stimulation was responsible. However, unlike in the mispositioning case, this patient’s FoG persisted when stimulation was turned off, and mapping of the leads suggests that they were in appropriate areas of the STN (Figure 1).

The FoG’s emergence weeks after lead activation, and persistence when stimulation was turned off, also argue against high-frequency stimulation as the cause of this patient’s symptoms. As higher frequencies helped control her painful dystonia and uncomfortable dyskinesias, lower frequencies (of 80 Hz, then 55 Hz) were not utilized until approximately nine months after FoG onset. At that time, the patient reported some improvement in FoG, but not to the degree seen in other cases where FoG was attributed to high-frequency stimulation [7].

Lastly, in terms of possible etiologies of the described refractory FoG, the possibility that freezing was due to overtreatment with dopaminergic therapy may be considered. This patient was on dual-dopaminergic therapy (Rytary, Requip) when the leads were activated [5,6]. Opposing this theory is the lack of a temporal pattern of FoG symptoms with medication intake, the persistence of FoG when off stimulation, and the relative stability of symptoms during the extended periods in which the patient was taking only one of the medications. A possible decrease in Rytary before FoG onset necessitates consideration that the initial incidence of FoG was due to insufficient dopaminergic coverage. However, this is similarly unlikely given the lack of any FoG before DBS and no clear relationship between the FoG and wearing-off or subsequent augmentation of the levodopa dosing to a level with a similar LEDD as the original pre-DBS Rytary.

In summary, the combination of findings that cannot be easily reconciled with any prior established or hypothetical etiology includes the acute onset of FoG with stimulation adjustment but a lack of response to lead inactivation and readjustment. Interpretation of this phenomenon is complicated by a complex subjective history and an incomplete understanding of the subacute and chronic effects of DBS. In speaking with the patient directly and in reviewing records, she consistently denied changes in her FoG when their device was off. However, she turned the device off infrequently immediately after implantation and even less so as time passed. This is because her painful toe dystonia, present in the off and low-frequency states, became increasingly problematic. Also clouding this unique feature of the case is uncertainty regarding how long these off-DBS periods were. The patient is unsure but believes she was “certainly” never off stimulation for more than a day, and likely not more than a “few hours” at a time.

The immediate positive effects of DBS are an often-advertised, dramatic example of the life-changing capabilities of modern medicine. The temporal patterns of symptoms after DBS cessation are perhaps less exciting and have not been investigated thoroughly. While early reports on STN-DBS suggested that up to three hours of discontinued stimulation may be needed to observe off effects [15], the return of tremors, bradykinesia, etc., is often quickly apparent clinically. Those with longer duration of disease may experience more rapid “washout” or cessation of DBS effects when stimulation is stopped [16]. More recent studies in globus pallidus internus DBS suggest therapeutic effects can last up to two days [17]. While this is a different anatomic location, it highlights the non-uniformity of DBS effects. An assumption that FoG follows the pattern of other PD symptoms in this regard may be incorrect.

If the positive effects of DBS take hours to days to subside, so too might the negative side effects take a significant amount of time to resolve. It is possible that the amount of time the patient tried turning the device off was insufficient for complete resolution of FoG. It was likely difficult for the patient and clinicians to tell if there was a dramatic difference in FoG on or off stimulation, as her FoG, though severe when present, was sporadic and not reproducible in a controlled manner.

If this hypothesis is correct, the FoG could be more easily explained by high-stimulus-induced dysfunction within the STN itself or in the surrounding gait-related pathways. Although reconstruction suggests the utilized contacts were in acceptable positions within the STN, the iatrogenic induction of FoG is too poorly understood to be sure that interference in these regions cannot cause freezing. If the initial higher frequencies were culpable, why did FoG persist with lowered frequencies? This could be due to continued stimulation of suboptimal regions or natural progression of disease in the significant time that elapsed between lead activation and the switch to low frequency. If contact positions were to blame, why did FoG not appear upon immediate activation of the leads? This remains unclear, but the patient noted gait issues, including “imbalance,” after activation. The initial settings may have caused only non-FoG gait issues, which resulted in FoG when adjusted.

A prolonged off-trial, up to several days, would be the most effective method to assess whether the FoG is stimulation-induced. The patient noted feeling she could not tolerate significant time off of DBS due to her dystonia. A simpler, alternative hypothesis is that this FoG represents a natural progression of the patient’s idiopathic PD. This is feasible, but the timing of onset with DBS is suspicious. Another structural consideration that could feasibly contribute to this unique timing of FoG onset is DBS-lead-associated gliosis/edema, differentially impacting the surrounding neural structures over time [18]. Assessment of these changes is virtually impossible outside of post-mortem examination.

Although the exact mechanism (aberrant stimulation, overstimulation, or mechanical/reactive lesioning) remains unclear, the timeline of symptom onset and density of gait-related pathways within the target region strongly suggest STN-DBS-induced freezing of gait (FoG) as the most likely etiology in this case. While disruption of the STN-MLR loop is one plausible contributor, it is not the sole mechanism by which interference in this area could impair gait. Multiple white matter pathways critical for locomotion course through the peri-stimulatory zone, including cortico-subthalamic projections (both hyper-direct and indirect pathways) and descending subthalamopontocerebellar tracts connecting the cerebellum to the STN via the pons [19].

Overall, this case illustrates the incomplete understanding of FoG as a complication of STN-DBS. It also highlights a need for more research into “off” effects of DBS. Continued reporting of similar cases, especially of the symptomatic timeline, stimulation parameters, and lead location within the STN via post-surgical imaging, is encouraged as this will allow for a more systematic appraisal of post-STN-DBS FoG in the future. A worthwhile next step will be to correlate stimulation settings and lead placement with clinical FoG outcomes using standardized assessments.

## Conclusions

This is a case of acute, post-STN-DBS FoG that began after the initial adjustment of stimulation parameters and persisted refractory to stimulation adjustments, stimulation on/off state, and dopaminergic medication. This perplexing history may be due to the lasting effects of DBS exceeding the short periods in which the patient turned off her device. Continued reporting of cases of post-DBS FoG, including lead location and stimulation parameters, is needed to identify patterns to inform future interventions.
